# Disrupted glutathione homeostasis in the pathogenesis of TTR-V30M amyloidosis

**DOI:** 10.1186/s40364-026-00970-8

**Published:** 2026-07-11

**Authors:** Anushree Bachhar, Gabriella Johannson, Melisnur Sahin, Sanduni Jayaweera, Malin Olsson, Intissar Anan, Anders Olofsson

**Affiliations:** 1 Department of Clinical Microbiology, Umeå University, Umeå, SE-901 87 Sweden; 2 Department of Public Health and Clinical Medicine, Umeå University, Umeå, SE-901 87 Sweden

**Keywords:** Pyroglutamate, ATTR, V30M, GSH homeostasis, *IDO1*

## Abstract

**Background:**

Transthyretin (TTR) amyloidosis is a progressive, life-threatening disorder caused by extracellular deposition of amyloid fibrils derived from the plasma protein TTR. Inherited forms are associated with destabilizing TTR mutations; however, recent findings indicate that amyloid formation in vivo may be promoted by disulfide bond formation between TTR subunits, suggesting oxidative stress as a potential contributor to protein misfolding and disease progression. Glutathione (GSH) is a central component of the antioxidant defense system, and disruption of GSH homeostasis can lead to the accumulation of pyroglutamate (PGA), which is detectable in plasma. Moreover, oxidative stress is frequently linked to inflammation, which may be reflected by increased indoleamine 2,3-dioxygenase 1 (*IDO1*) activity, observed as an elevated plasma kynurenine/tryptophan ratio.

**Methods:**

Plasma levels of PGA, kynurenine, and tryptophan were quantified by liquid chromatography-mass spectrometry in cohorts comprising healthy TTR wild-type controls, asymptomatic carriers of the TTR-V30M mutation, and symptomatic patients with TTR-V30M amyloidosis.

**Results:**

Symptomatic individuals had significantly elevated plasma PGA levels compared with both asymptomatic carriers and age-matched healthy controls, consistent with impaired GSH homeostasis. In parallel, the kynurenine/tryptophan ratio was increased in symptomatic TTR-V30M carriers, supporting inflammatory activation in manifest disease.

**Conclusions:**

These findings identify disrupted GSH homeostasis and inflammatory activation as metabolic features associated with symptomatic TTR-V30M amyloidosis in vivo, supporting a link between redox imbalance and disease manifestation. Strategies aimed at restoring antioxidant homeostasis and limiting inflammatory oxidative stress may therefore warrant further investigation as approaches to delay onset or slow disease progression.

**Supplementary Information:**

The online version contains supplementary material available at 10.1186/s40364-026-00970-8.

## Introduction

Transthyretin (TTR) amyloidosis (ATTR) is a progressive, life-threatening disorder characterized by the extracellular deposition of amyloid fibrils derived from the TTR protein [1]. Symptoms frequently include progressive polyneuropathy, gastrointestinal disturbances, and cardiac dysfunction [2]. Today, more than 130 mutations in TTR have been linked to TTR amyloidosis, where the TTR-V30M as well as TTR-V122I represent the two most common variants [3]. Notably, wild-type TTR is also associated with amyloidosis and is predominantly linked to cardiac manifestations [4]. Wild-type TTR amyloidosis primarily affects the heart, and although often asymptomatic, amyloid deposits can be detected in approximately 25% of men over 80 years of age [5], making TTR amyloidosis one of the most common amyloid disorders.

The hereditary form of TTR amyloidosis is associated with an earlier age of onset; however, both the age of onset and disease penetrance vary considerably among different mutations and across endemic regions [6, 7]. In Sweden, the most common variant is TTR-V30M, which is also present in Portugal, Brazil, and Japan. While TTR-V30M carriers in the Portuguese, Brazilian, and Japanese populations share a common ancestral origin, the same mutation in Sweden has arisen independently [8]. In Sweden, the average age of onset is approximately 56 years, with a lifetime risk of around 50% [9, 10]. The prevalence of TTR amyloidosis is also influenced by sex, with symptoms of wild-type TTR amyloidosis occurring almost exclusively in men [11, 12]. This skewed distribution is also observed in the inherited forms of the disease, where around 70% of the affected individuals are males [13, 14]. TTR is a tetrameric protein with a molecular weight corresponding to 55 kDa; it is involved in the transport of retinol-binding protein as well as the transport of thyroxine hormone [15, 16]. The conversion of TTR from its native structure to the amyloid form requires partial denaturation [17–19]. Destabilizing mutations facilitate this initial transition, and the increased rate of conversion to a pathological state likely contributes to the earlier age of onset compared with wild-type TTR amyloidosis. Although reduced protein stability promotes amyloid formation, it alone cannot fully explain disease onset, as pathology is also influenced by external factors. Notably, differences in the timing of disease onset have been observed even among monozygotic twins, and in a case report, one sibling remained symptom-free for up to 13 years after the other had already developed the disease [20]. Such variation in clinical presentation strongly indicates that, beyond protein stability and genetic factors, additional influences contribute to disease onset and progression.

In a previous study, a pathological non-native fold of TTR, termed NNTTR, was identified in plasma from TTR-V30M carriers [21]. Higher levels of NNTTR were observed in symptomatic disease and pre-treatment levels associated with clinical response to TTR-stabilizing drug tafamidis [21]. Through characterization of the NNTTR species in human plasma, we recently demonstrated that its formation is strongly dependent on a non-native disulfide bond formed between Cys10 on two TTR subunits within the tetramer [22]. This modification uniquely induces structural rearrangements associated with amyloidogenic conversion, in contrast to other post-translational modifications on Cys10, such as, S-cysteinylation or S-glutathionylation, that do not significantly alter the native fold [22]. A Cys10–Cys10′ disulfide bond is abundantly present in ex vivo-isolated TTR-V30M amyloid fibrils [23]. Moreover, replacement of Cys10 with serine in a TTR-V30M-based mouse model has been shown to prevent amyloid formation [24]. The present study is therefore based on the hypothesis that disulfide formation is critical in the pathogenesis, and to emphasise the presence of a disulfide bond, NNTTR will hereafter be referred to as dsNNTTR. It should nevertheless be stated that although the above studies indicate a critical role of disulfide linkage in the pathology of TTR-V30M amyloidosis, other mechanistic explanations cannot be excluded.

The conversion of two thiol groups into a disulfide bond represents an oxidation reaction and therefore requires an oxidizing agent acting as an electron acceptor. Although many molecules can function as oxidants, in vivo reactive oxygen species (ROS) and reactive nitrogen species (RNS) are of relevance.

Free radicals are generated through several different pathways, e.g., during mitochondrial aerobic respiration, approximately 1–2% of electrons may escape the electron transport chain, and an estimated 2% of the oxygen consumed by mitochondria contributes to ROS formation, primarily the superoxide radical [25]. These species contribute to oxidative and nitrosative stress by promoting protein nitration and nitrosation reactions, thereby affecting enzyme activity and cellular signalling pathways. Oxidative stress has been implicated in the majority of amyloid disorders, and recent reviews provide comprehensive overviews of its role in TTR amyloidosis [26]. Free radicals are characterized by exceptionally high reactivity, primarily due to the presence of at least one unpaired electron in their outer orbital. This unpaired electron creates an energetically unstable state, driving the radical to rapidly interact with surrounding molecules to achieve electron pairing.

Both ROS and RNS are continuously generated in vivo as part of normal cellular metabolism and at physiological levels, they serve important signalling and regulatory functions; however, excessive production or insufficient clearance leads to oxidative and nitrosative stress, which can damage lipids, proteins, and nucleic acids. Among reactive oxygen species, the superoxide anion (O₂•⁻) is primarily generated as a by-product of mitochondrial oxidative phosphorylation when electrons leak from the electron transport chain. It is also deliberately produced by immune cells through NADPH oxidase as part of the respiratory burst used to eliminate pathogens. Superoxide is rapidly converted into hydrogen peroxide (H₂O₂), by superoxide dismutase [27]. Despite its lower reactivity, H₂O₂ becomes highly damaging in the presence of transition metals such as iron or copper, where it can generate the highly reactive hydroxyl radical (•OH) [28]. Reactive nitrogen species arise primarily from the metabolism of nitric oxide (NO•), a signalling molecule synthesized from arginine by nitric oxide synthases [29]. Nitric oxide has vital roles in vasodilation, neurotransmission, and immune regulation. It is relatively unreactive, but upon interaction with superoxide, it may result in peroxynitrite (ONOO⁻), which is associated with cellular injury and inflammation. Additional reactive nitrogen species include nitrogen dioxide (NO₂•) and dinitrogen trioxide (N₂O₃), which arise from secondary reactions involving nitric oxide and oxygen [30]. Another reaction that generates free radicals is the purine metabolism, where xanthine oxidase catalyses the conversion of hypoxanthine to xanthine and subsequently to uric acid, and during its re-oxidation, molecular oxygen is reduced to superoxide [31].

Although both ROS and RNS are essential for many cellular functions, they require strict regulation by antioxidant systems. Oxidative stress refers to a disruption in the balance between oxidant molecules and antioxidant defences, with a shift toward oxidant predominance. This condition may arise from increased production and accumulation of reactive species or from reduced availability or function of antioxidant mechanisms. In the Swedish population, the inherited form of TTR amyloid accumulation is typically asymptomatic until middle age or later, indicating that age-related changes may contribute to disease onset. Among these changes is an age-dependent increase in both ROS and RNS [32–34].

Notably, amyloid deposits in ATTR patients correlate with markers of lipid peroxidation, such as 4‑hydroxy‑trans‑2‑nonenal (HNE) adducts [35] and oxidative DNA damage indicated by 8‑hydroxy‑2′deoxyguanosine (8‑OhdG) [34]. Animal studies indicate a beneficial effect of the flavonoid curcumin on TTR amyloidosis, where it significantly reduced ATTR aggregate deposition in the dorsal root ganglia [36]. Using a well-known β‑blocker with potent antioxidant properties, a lowering of both HNE and 8‑OhdG adducts, alongside a decrease in total TTR deposits, has been noted [37].

**Fig. 1:**
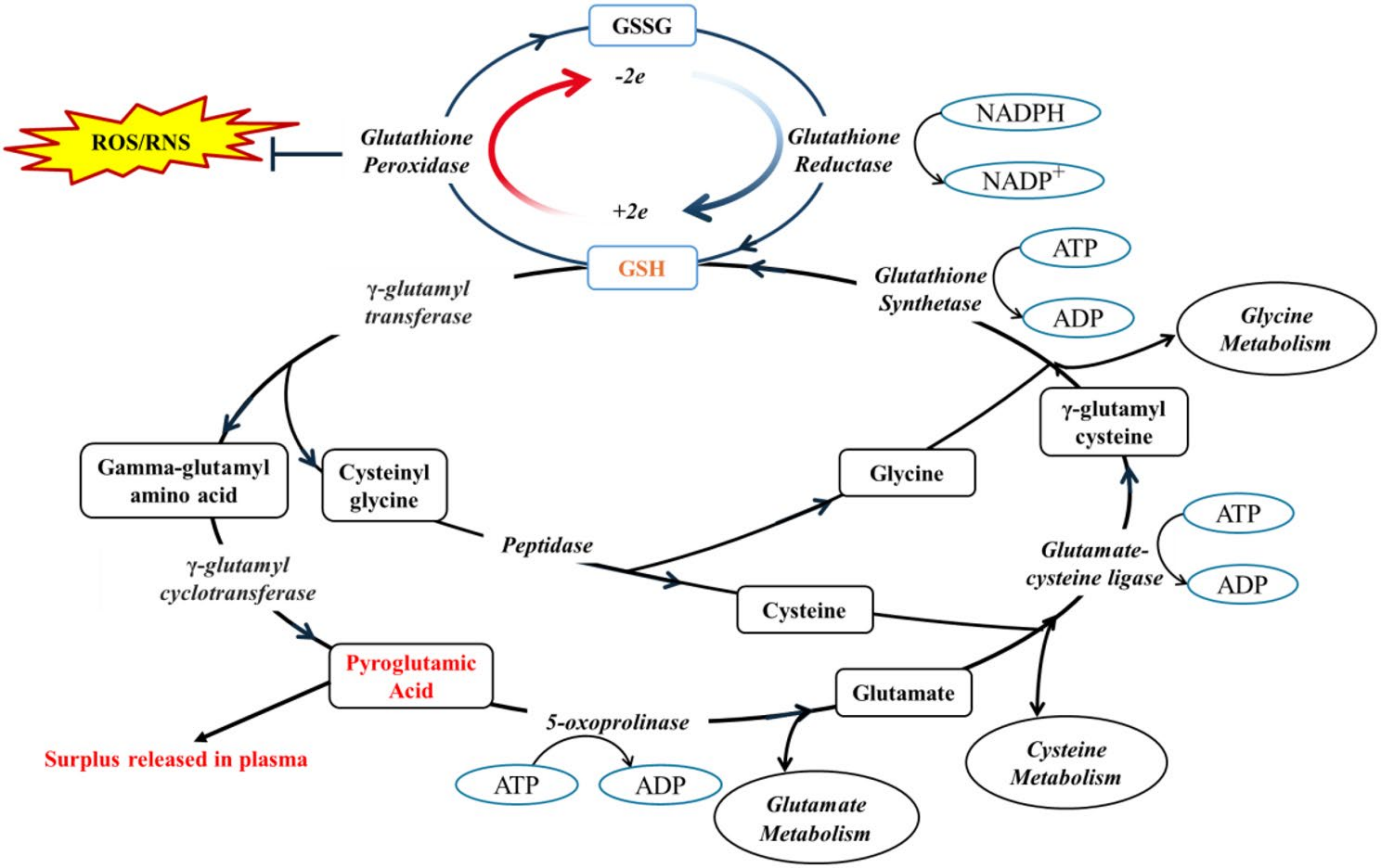
Biochemical pathway model of glutathione metabolism. Schematic illustration depicting both glutathione (GSH) synthesis and its role in antioxidant defense. The upper cycle represents glutathione recycling, with metabolic intermediates and enzymes arranged clockwise, beginning with reduced glutathione (GSH), followed by glutathione peroxidase (EC 1.11.1.9), NADPH-dependent glutathione reductase (glutathione disulfide reductase; EC 1.8.1.7), and oxidized glutathione (GSSG). The lower cycle illustrates glutathione synthesis and regeneration, with intermediates and enzymes arranged counterclockwise, beginning with γ-glutamyltransferase (EC 2.3.2.2), peptidase (alanyl aminopeptidase; EC 3.4.11.2), γ-glutamyl cyclotransferase (glutathione-specific γ-glutamyl cyclotransferase 2; EC 4.3.2.7), ATP-dependent 5-oxoprolinase (EC 3.5.2.9), ATP-dependent γ-glutamate-cysteine ligase (EC 6.3.2.2; comprising catalytic and modifier subunits), and ATP-dependent glutathione synthetase (EC 6.3.2.3). Bidirectional arrows connecting glutamate, cysteine, and glycine to amino acid metabolism indicate that these substrates can enter or exit the glutathione pathway depending on cellular metabolic demands. The pathway model was curated based on the Kyoto Encyclopedia of Genes and Genomes (KEGG) glutathione metabolism map (KEGG: hsa00480)

Physiologically, ROS and RNS are detoxified by enzymatic systems such as catalase, thioredoxin, and superoxide dismutase [38]. In this context, however, the thiol antioxidant glutathione (GSH) represents a dominant defence system with the capacity to neutralize a broad range of both ROS and RNS [39]. This neutralizing effect is mediated by a family of glutathione peroxidase enzymes (GPx). During this process, GSH is oxidized to its disulfide-linked form, glutathione disulfide (GSSG). GSSG can subsequently be reduced back to GSH by glutathione reductase, which is coupled to NADPH as the electron donor. As a result, GSH is continuously recycled and available for repeated detoxification cycles [40]. GSH is a tripeptide synthesized intracellularly through two sequential, ATP-dependent enzymatic steps. In the first step, glutamate and cysteine are ligated by γ-glutamylcysteine synthetase (also known as glutamate–cysteine ligase, GCL) to form γ-glutamylcysteine, utilizing one molecule of ATP. This reaction forms an unusual γ-peptide bond between the γ-carboxyl group of glutamate and the amino group of cysteine. In the second step, glycine is added to the carboxyl group of cysteine by glutathione synthetase, again consuming one molecule of ATP, to produce the final tripeptide glutathione (GSH), see Fig. 1. Under normal conditions, glutathione levels are tightly regulated to ensure both adequate antioxidant capacity and controlled metabolic flux through its biosynthetic pathway. The first and rate-limiting step of glutathione synthesis, the formation of γ-glutamylcysteine from glutamate and cysteine, is normally subject to negative feedback inhibition by glutathione itself [41]. When intracellular glutathione levels are sufficient, this feedback mechanism suppresses further synthesis, preventing excessive accumulation of pathway intermediates. However, when glutathione metabolism becomes impaired, intracellular GSH levels decline, and this regulatory control is lost. As glutathione levels decrease, feedback inhibition of γ-glutamylcysteine synthesis is lifted, leading to increased production of γ-glutamylcysteine. If the downstream steps of glutathione synthesis cannot keep pace, due to either limited glycine availability, reduced enzymatic capacity, or energy depletion, this intermediate will accumulate. Excess γ-glutamylcysteine is metabolically unstable and is diverted into an alternative pathway, where it is converted into PGA. Under normal conditions, PGA is a transient intermediate that is rapidly converted back to glutamate by the enzyme 5-oxoprolinase, allowing the cycle to proceed efficiently [42]. This conversion is ATP-dependent, requiring sufficient cellular energy to proceed at a normal rate. In the context of impaired glutathione metabolism, two converging factors promote PGA accumulation. First, increased upstream production elevates the amount of PGA generated. Second, metabolic stress, often accompanied by ATP depletion, limits the cell’s capacity to reconvert PGA to glutamate via 5-oxoprolinase, resulting in excess PGA being released into the plasma [42].

Elevated PGA therefore represents a biochemical signature of disrupted glutathione homeostasis. Its accumulation reflects impaired glutathione regeneration, loss of regulatory control within the γ-glutamyl cycle, and metabolic conditions that favour diversion of intermediates away from productive glutathione synthesis. Consequently, PGA formation constitutes an “overflow” pathway that becomes prominent only when normal glutathione homeostasis is compromised, making it a valuable marker of impaired cellular antioxidant capacity and metabolic stress. A schematic illustration depicting GSH synthesis, its oxidation to GSSG, and the subsequent reduction of GSSG back to GSH using NADPH is shown in Fig. 1.

Glutathione depletion can occur through several mechanisms, including insufficient availability of precursor amino acids (particularly cysteine), increased utilization during oxidative stress, low selenium, and impaired recycling from its oxidized form, GSSG. Inflammation is in this context closely associated with oxidative stress and may therefore lead to increased GSH consumption. Notably, several chronic inflammatory conditions have been linked to elevated PGA levels, which can be attributed to a higher consumption of GSH [43–45].

To elucidate the potential impact of TTR amyloidosis–associated oxidative stress on GSH homeostasis, we quantified plasma PGA levels in three well-defined cohorts aged 55 years and older: asymptomatic carriers of the TTR-V30M mutation, symptomatic carriers with confirmed TTR amyloidosis (TTR-V30M patients), and healthy controls with the wild-type TTR genotype. The results revealed a significant increase in plasma PGA levels in TTR-V30M patients, whereas no significant elevation was observed in asymptomatic carriers or healthy controls.

Oxidative stress can be mediated by several different mechanisms but is frequently linked to inflammation. Interestingly, we also show that kynurenine, a metabolite derived from tryptophan following inflammatory activation of *IDO1* [46–48], is elevated in TTR-V30M patients but not in age-matched healthy controls.

Taken together, we propose a model in which increased oxidative stress in TTR-V30M carriers, potentially promoted by misfolded TTR, contributes to reduced GSH availability [49, 50]. This may limit the capacity to neutralize ROS and RNS, thereby facilitating further formation of dsNNTTR. Restoring GSH homeostasis could therefore help counteract redox imbalance and represents a potential therapeutic or prophylactic strategy that warrants further investigation.

## Methodology

### Plasma sample collection

To minimise the effect of environmental factors impacting overall metabolomics among individuals, all patients were chosen from the Northern Sweden cohort. In total, 105 peripheral blood samples were collected from three groups of older adults (≥ 55 years): healthy controls (wild-type TTR) *n* = 26 (16 male; 10 female), asymptomatic TTR-V30M gene carriers *n* = 30 (10 male; 20 female), and patients with ATTR-V30M amyloidosis *n* = 47 (30 male; 17 female) (Supplemental Information: Table S1). The identification criteria and protocol for selection of TTR V30M patients, TTR V30M carriers, and healthy controls are provided in Supplemental Information under the heading cohort selection criteria. Briefly, the patient’s diagnosis was confirmed by biopsy, stained for amyloid using Congo red [51] and/or 99mTc-DPD, 3,3-diphosphono-1,2-propanodicarboxylic acid scintigraphy, and TTR mutation status was verified by PCR-based sequencing [52]. None of the included individuals had diagnosed liver or renal dysfunction at the time of sampling or were reported as malnourished.

At age 55 years and older, the majority of symptomatic TTR-V30M gene carriers in northern Sweden are male. Accordingly, we observed more male patients and fewer asymptomatic male carriers, whereas among females, this pattern was reversed, with more asymptomatic carriers than patients. An equal number of individuals of each sex in each group could therefore not be maintained, and both male and female participants were thus included in the analysis. However, biomarker differences, represented by plasma PGA levels and the kynurenine/tryptophan (Kyn/Trp) ratio, were consistent across sexes.

Volunteers who agreed to donate plasma were instructed to fast overnight for 12 h, and plasma was collected in the morning. This procedure was used to minimize potential interference from diet and medication.

Blood was collected in EDTA tubes and centrifuged at 1,600 × g for 16 min at room temperature to separate the plasma, which was aspirated and stored at − 80 °C.

Plasma samples were thawed in batches of approximately 20 to minimize thaw time. For each sample, 120 µL of plasma was aliquoted into two round-bottom 96-well plates, kept on dry ice, sealed with plastic film, and stored at − 80 °C until LC-MS/MS sample preparation and analysis. To prevent potential run-order effects on metabolite levels, sample placement within the plates was randomised among carriers, affected individuals, and controls. In addition, sample distribution and well positions were balanced across the two plates to further minimize analytical bias.

### Absolute quantification of pyroglutamic acid

Targeted quantification of PGA by LC-MS was performed with a specific protocol and performed according to [53–55] at the Swedish Metabolomics Center in Umeå, Sweden. Briefly, metabolites in plasma (100 µL) were extracted with 90% methanol (900 µL), and proteins were precipitated at -20 °C for 2 h, followed by centrifugation for 10 min at 4000 rpm at 4 °C. Supernatant (20 µL) was collected and dried in a 96-well plate under nitrogen flow at 40 °C. Upon analysis, samples were resuspended in 200 µL water with 50 nM pyroglutamic acid-^13^C_5_ (MedChemExpress, Monmouth Junction, NJ, USA) as an internal standard. The chromatographic separation was performed on an Agilent 1290 Infinity UHPLC-system (Agilent Technologies, Waldbronn, Germany). 5 µL of each sample was analysed on a Acquity UPLC HSS-T3 column (50 × 2.1 mm, 1.8 μm, Waters, MA, USA) by using gradient elution of 0.1% formic acid (*v*/*v*) in water as mobile phase A and 0.1% formic acid (*v*/*v*) in acetonitrile as mobile phase B, with a flow rate of 0.4 mL/min with the following gradient: 0–0.5 min (0% B), 0.8 min (15% B), 1.2 min (80% B), 1.4 min (80% B), 1.5 min (0% B), 2 min (0% B). Column and autosampler were kept at 40 °C and 4 °C, respectively. The compounds were detected with a 6495 C Triple Quadrupole LC/MS (Agilent) mass spectrometer equipped with a jet stream electrospray ion source operating in positive ion mode. Multiple Reaction Monitoring (MRM)-transitions for PGA were as follows: PGA MRM transition: 130.0->84.1; MS polarity: ‘+’. For internal standard (IS): PGA^13^C_5_; MRM transition (IS)135.0->88.0; MS polarity (IS): ‘+’.

### 3-Nitrophenyl hydrazine based quantification of kynurenine and tryptophan

The sample preparation 3-Nitrophenyl hydrazine (NPH) derivatization method was carried out as described in [56, 57]. The extraction mixture content was 90% methanol and an internal standard for Kynurenine and tryptophan (Sigma-Aldrich, St. Louis, MO, USA). Absolute quantification was performed using isotope-labelled internal standards and validated calibration curves to ensure analytical accuracy and reproducibility. The sample preparation for NPH derivatization and extraction was carried out by the Swedish Metabolomics Centre. Methanol HPLC-grade, and Acetonitrile HPLC-grade were obtained from Fischer Scientific (Waltham, MA, USA). H_2_O, Milli-Q, and IS: Kynurenine-D6 and Tryptophan-D8 were obtained from Sigma-Aldrich (St. Louis, MO, USA). IS: PGA^13^C_5_ was obtained from MedChemExpress (Monmouth Junction, NJ, USA).

The MRM values are as follows: Kynurenine: 344.1->309.0, MS polarity:‘+’, Kynurenine-D6 (IS) 350.1->178, MS polarity (IS) ‘+’ ; Tryptophan 340.1->323.0, MS polarity : ‘+’, Tryptophan-D8 (IS) 348.1->209.0, MS polarity (IS): ‘+’.

The metabolic data processing was performed using Agilent Masshunter Quantitative Analysis for QQQ version 12.0, build 12.0.893.1 (Agilent Technologies Inc., Santa Clara, CA, USA). For PGA, concentrations were calculated from the ratio of endogenous metabolite to isotopically labelled internal standards, compared against calibration curves of increasing endogenous standard concentrations. Concentrations were expressed as nM and then recalculated to µM in plasma. The signal-to-noise ratio (S/N) for PGA for every sample was > 10, LOD/LOQ: ≥ 0.3. Similarly, separate calibration curves were prepared for both Kynurenine and tryptophan for absolute quantification with Agilent Masshunter Quantitative Analysis for QQQ version 12.0, built on 12.0.893.1 software. (Agilent Technologies Inc., Santa Clara, CA, USA) at Swedish Metabolomics Centre, Umeå, Sweden. The S/N for both Kynurenine and tryptophan for every sample was > 10, with LOD/LOQ: ≥ 0.3 for both tested metabolites.

### Preparation of TTR-V30M

Recombinant TTR-V30M variant expressed in *E. coli* was obtained from Alexotech (Umeå, Sweden). Before use, the protein was denatured in 6 M urea and supplemented with 2 mM tris(2-carboxyethyl)phosphine (TCEP, Sigma-Aldrich, Solna, Sweden) at pH 7.4 to obtain complete reduction and dissolution of potential aggregates. The samples were subsequently purified by size-exclusion chromatography using a Superose 6 10/300 column (Cytiva, Uppsala, Sweden) equilibrated with PBS (pH 7.5).

To elucidate the effect of nitrosylation 8 µM of recombinant TTR-V30M was mixed with S-nitrosylglutathione (Sigma-Aldrich, St. Louis, MO, USA) in a serial dilution from 400 to 0 µM, followed by incubation at room temperature for 2 h, followed by removal of S-nitrosylglutathione (GSNO) through a buffer exchange using a PD10 column (Bio-Rad, Solna, Sweden). In parallel, samples were incubated with the corresponding concentration of H_2_O_2,_ (Sigma-Aldrich, St. Louis, MO, USA) as well as a single control incubated with the reducing agent β-mercaptoethanol (Thermo Scientific, Stockholm, Sweden). The samples were then incubated at 37 °C for either 30 min–2 h.

For preparation of reduced controls, 100 µM β-mercaptoethanol was added and incubated under identical conditions. The molecular masses of all protein species were monitored by liquid chromatography–time-of-flight mass spectrometry (LC/MS TOF 6230B, Agilent, Santa Clara, CA, USA) using the MassHunter Qualitative Analysis 12.0 software (Agilent, Santa Clara, CA).

### SDS–PAGE

TTR-V30M samples were combined with a non-reducing 4×SDS sample buffer (pH 6.8; Bio-Rad, Solna, Sweden) and heated to 98 °C for 10 min to achieve complete protein denaturation. The proteins were then separated on 4–20% gradient precast polyacrylamide gels (Mini-PROTEAN TGX, Bio-Rad, Solna, Sweden). Following electrophoresis, protein bands were detected by staining with Coomassie Brilliant Blue R-250 (Merck, Stockholm, Sweden) using conventional staining protocols. The molecular mass of TTR was independently confirmed by time-of-flight liquid chromatography-mass spectrometry (LC/MS TOF 6230B, Agilent, Santa Clara, CA, USA).

### Statistical analysis

**Fig. 2:**
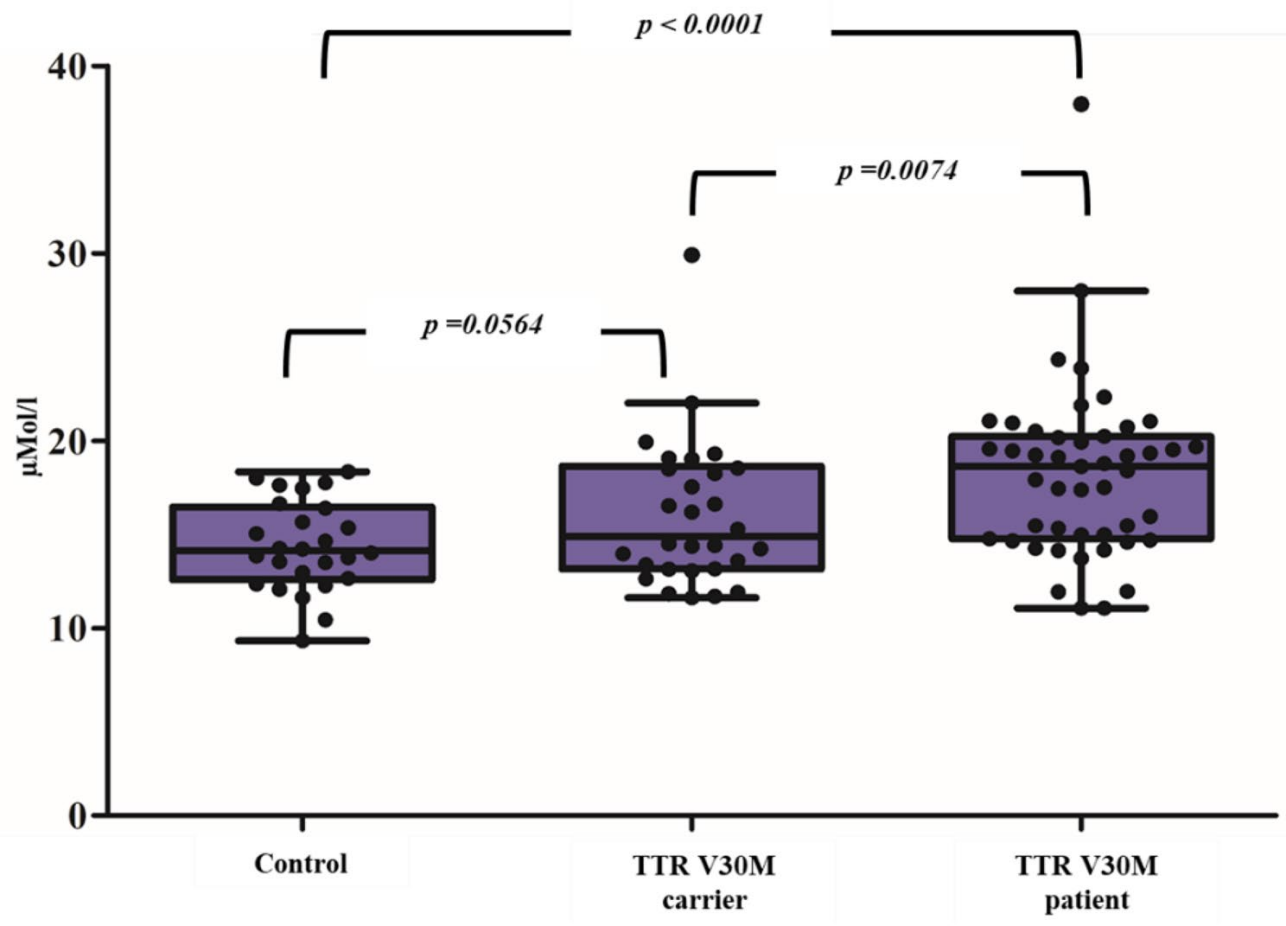
Plasma pyroglutamic acid levels in healthy controls, asymptomatic TTR-V30M carriers, and symptomatic TTR-V30M patients. Boxplots with whiskers (Turkey) show pyroglutamic acid concentrations (µmol/L) in the three study groups: healthy controls, asymptomatic TTR-V30M carriers, and symptomatic TTR-V30M patients. The line within each box indicates the median, and the box represents the interquartile range (IQR). Whiskers represent the Q1 IQR percentile 25*1.5 and Q3 IQR percentile 75*1.5 of pyroglutamic acid levels, *p* values (one-tailed) were calculated using an unpaired non-parametric Mann-Whitney U test to compare the study groups. Statistical significance was defined as *p* < 0.05. Associated statistical analysis data are provided in Supplementary Information, Tables S2 and S3. The graph was created using GraphPad Prism 5 Statistical software for Windows (GraphPad Software, San Diego California, USA)

**Fig. 3:**
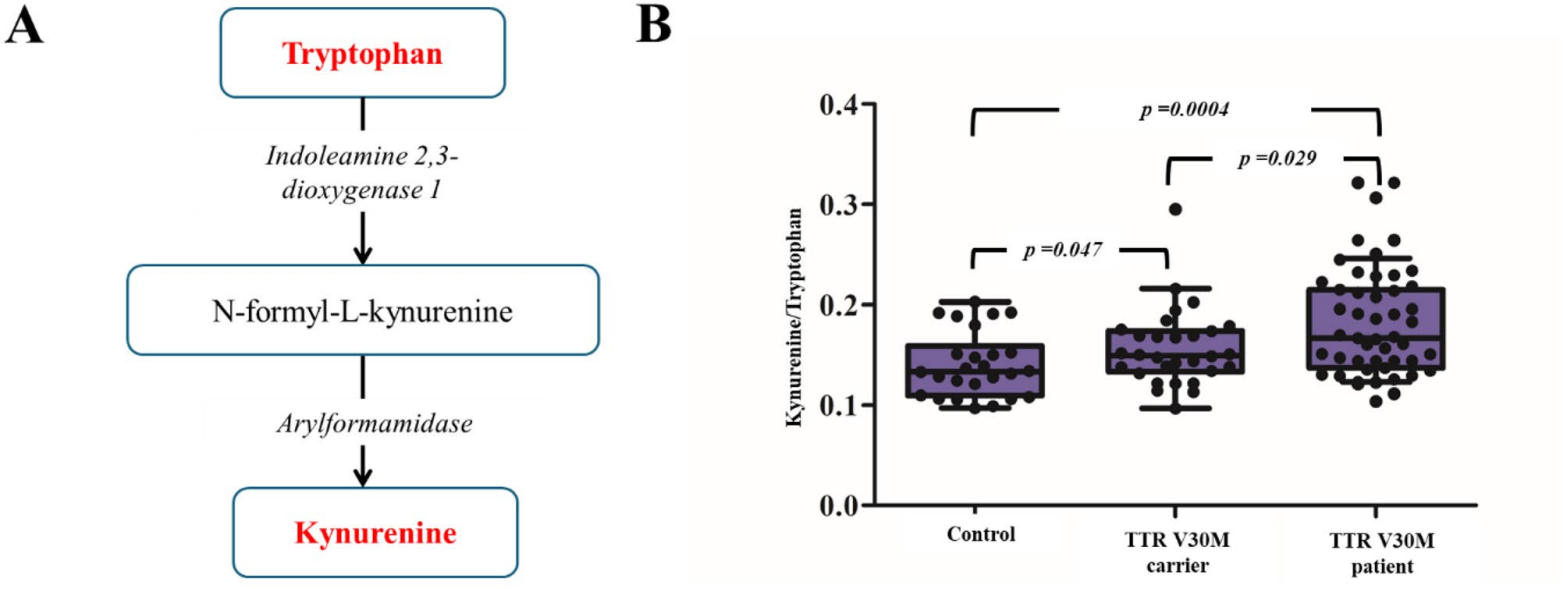
Kynurenine pathway and plasma kynurenine/tryptophan ratio in TTR-V30M amyloidosis. (**A**) Schematic representation of the kynurenine pathway of tryptophan metabolism. Metabolites quantified in this study are highlighted in red. Indoleamine 2,3-dioxygenase (IDO ; EC 1.13.11.52) and arylformamidase (AFMID; EC 3.5.1.9) are indicated. The pathway scheme was curated from KEGG tryptophan metabolism pathway hsa00380. (**B**) Plasma kynurenine/tryptophan ratio in healthy controls, asymptomatic TTR-V30M carriers, and symptomatic TTR-V30M patients. Boxplots show medians and interquartile ranges; whiskers represent the Q1 IQR percentile 25*1.5 and Q3 IQR percentile 75*1.5 of Kyn/Trp levels. *p* values were calculated using one-tailed unpaired Mann–Whitney *U* tests. Statistical significance was defined as *p* < 0.05. Associated statistical analyses are provided in Supplemental Information, Tables S2 and S3. GraphPad Prism 5 Statistical software for Windows (GraphPad Software, San Diego California, USA)

The statistical analyses and figures (Figs. 2 and 3B) were carried out using GraphPad Prism 5 Statistical software for Windows (GraphPad Software, San Diego California, USA). The analyses were primarily performed using nonparametric methods (Kruskal–Wallis and Mann–Whitney U tests) due to non-Gaussian data distribution. Both one- and two-tailed analyses were conducted and yielded consistent results. For group-based comparisons, Kruskal-Wallis analysis of variance (ANOVA) and non-parametric Mann–Whitney U tests for pairwise group comparisons. A summary of the Kruskal–Wallis and Mann–Whitney U test results with detailed statistical analyses is provided in Supplemental Information: Tables S2 and S3.

### Biomarker validation

The Receiver Operating Characteristic (ROC) curve analysis was performed using MetaboAnalyst 6.0 online tool/ Biomarker analysis. The results are tabulated in Supplemental Information: Table S4, and the Area under the curve (AUC) is presented in Supplemental Information, figure S1A-C.

## Results

### Symptomatic carriers of the TTR-V30M mutation display elevated plasma PGA levels.

Plasma PGA levels, reflecting glutathione (GSH) metabolic homeostasis, were analysed in three groups represented by: healthy controls with the wild-type TTR genotype, asymptomatic carriers of the TTR-V30M mutation, and symptomatic patients with TTR-V30M amyloidosis. PGA levels were increased by approximately 32% in symptomatic individuals compared with healthy controls (CV = 25.45% and 16.55%, respectively; Mann-Whitney U test, *p* < 0.0001) (Table 1) using both one-tailed and two-tailed analysis. A similar statistically significant (both one and two-tailed) increase was observed when symptomatic patients were compared with asymptomatic carriers (CV = 40.67%), with an approximately 25% elevation in PGA levels (*p* < 0.05) (Table 1; Fig. 2). The majority of symptomatic V30M patients had higher PGA levels than both healthy controls and asymptomatic V30M carriers, consistent with a disease-associated increase in PGA.

Given that the asymptomatic TTR-V30M carriers were 55 years or older, it is reasonable to assume that this group also includes individuals in a prodromal stage of disease, which could explain the higher CV. Nevertheless, on a group level, PGA in asymptomatic TTR-V30M carriers did not differ significantly from the healthy control (*p* > 0.05; Fig. 2). Detailed statistical analyses are provided in Supplemental Information, Tables S2 and S3.

**Table 1:** Percentage change of plasma pyroglutamic acid levels and their distribution among the groups

Group	Percentage change (%) in metabolites	The percentage of the population having a higher value compared with the median of the divisor group
**Pyroglutamic Acid**		
Carrier V30M/ Healthy control	5.55	63.33
Patients V30M/ Healthy Control	31.89	89.36
Patients V30M/ Carrier V30M	24.97	74.40

The comparative analysis results found in columns 2 and 3 were carried out by using the median value of each group. The detailed statistical analysis could be found in Supplemental Information

### An increased kynurenine/tryptophan (Kyn/Trp) ratio correlates with disease

The kynurenine pathway is a major route of tryptophan metabolism in humans, in which the enzyme indoleamine 2,3-dioxygenase (*IDO1*) converts tryptophan to kynurenine via the intermediate N-formyl L-kynurenine, followed by deformylation mediated by arylformamidase [58–60], for a schematic illustration, see Fig. 3A. Interestingly, *IDO1* is closely associated with chronic inflammatory responses [61, 62] and increased *IDO1* activity through upregulation, then alters the ratio between kynurenine and tryptophan [63]. Using a conservative two-tailed analysis, we found a significant increase in the Kyn/Trp ratio in symptomatic TTR-V30M patients compared with healthy controls (*p =* 0.0009; Supplemental Information, Table S3), supporting inflammatory activation in manifest disease. The Kyn/Trp ratio was also directionally higher in symptomatic patients than in asymptomatic V30M carriers, although this comparison did not reach statistical significance in the two-tailed analysis (*p* = 0.0594). Similarly, the comparison between asymptomatic V30M carriers and healthy controls did not reach statistical significance in the two-tailed analysis (*p* = 0.0954; Supplemental Information, Table S3). Because *IDO1*-associated inflammatory activation would be expected to increase, rather than decrease, the Kyn/Trp ratio [46–48], a one-tailed analysis was also performed. This further supported the statistical difference between symptomatic patients and healthy controls (*p* = 0.0004). It also indicated directional differences between symptomatic patients and asymptomatic V30M carriers (*p* = 0.0297), as well as between asymptomatic V30M carriers and healthy controls (*p* = 0.0477; Fig. 3B). Collectively, these findings may suggest that the asymptomatic carrier group includes individuals with early biochemical changes who are closer to disease onset. Although of potential prognostic interest, this interpretation should be considered exploratory and requires confirmation in independent longitudinal cohorts.

**Table 2:** Percentage change of plasma kynurenine/tryptophan levels to compare the relative upregulation of *IDO1* enzyme

Group	Percentage change (%) in metabolites	The percentage of the population having a higher value compared with the median of the divisor group
**Kynurenine/Tryptophan**		
Carrier V30M/ Healthy control	12.03	80
Patients V30M/ Healthy Control	25.20	82.97
Patients V30M/ Carrier V30M	11.97	63.82

The comparative analysis results found in columns 2 and 3 were carried out by using the median value of each group. The detailed statistical analysis could be found in Supplemental Information

### Nitrosylation of cysteine 10 of TTR-V30M promotes dsNNTTR formation

**Fig. 4:**
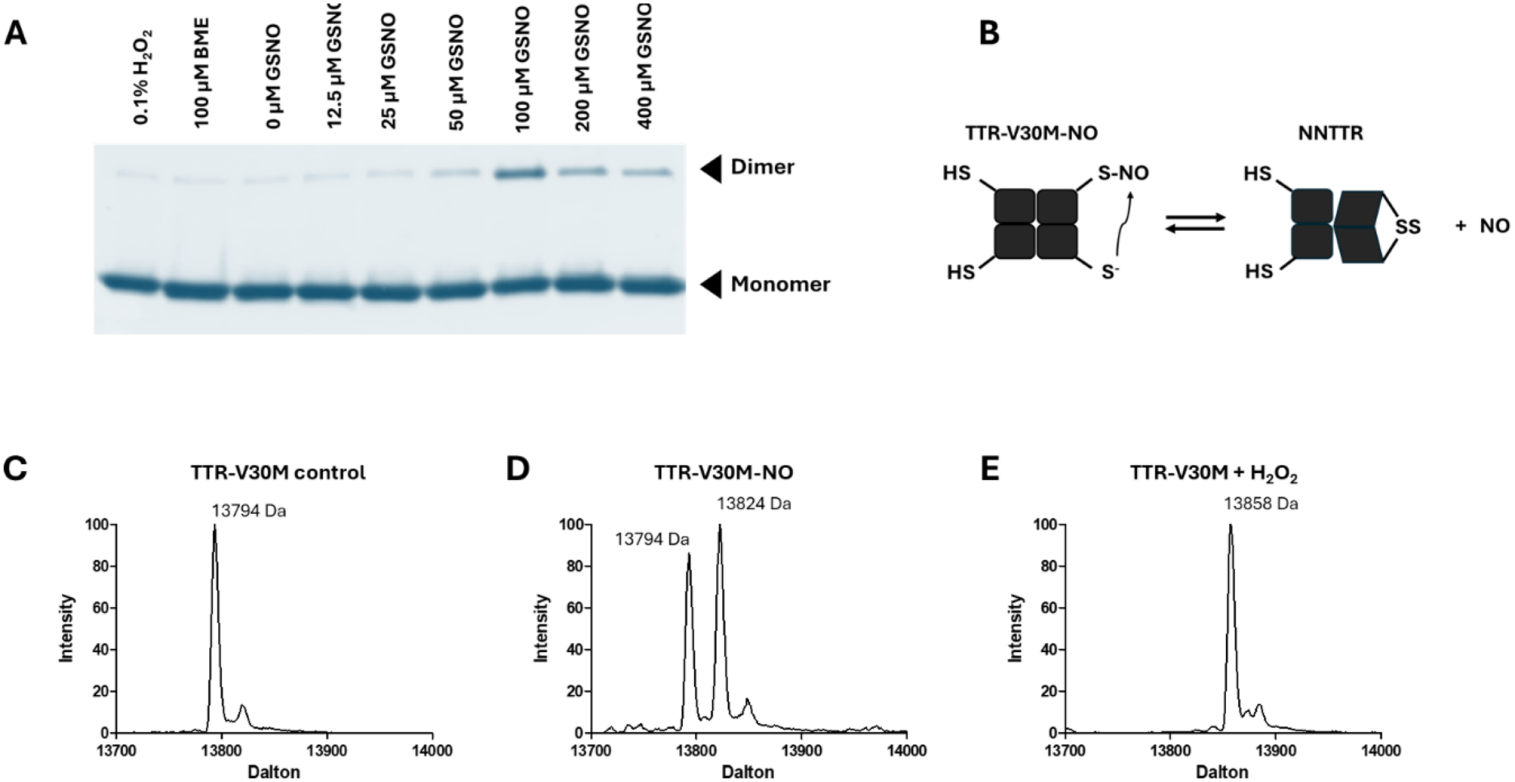
(**A**) Non-reducing SDS–PAGE analysis following incubation of 8 µM recombinant TTR-V30M in PBS (pH 7.4) in the presence of increasing concentrations of the oxidizing agent S-nitrosylglutathione (GSNO). A sample containing 100 µM β-mercaptoethanol and 0.1% H_2_O_2,_ respectively are included as a control. S-nitrosylation occurs on the free single cysteine residue located in position 10, of TTR and is observed as a + 30 Da mass adduct. S-nitrosylation facilitates subsequent disulfide bond formation through reaction with a thiolate on the adjacent protein subunit. The extent of labelling is thus critical, as both insufficient and excessive modification reduce overall conversion, with an optimal effect, observed at 100 µM GSNO under these conditions, seen as the most prominent dimeric band. A densitometric analysis of the relative ratio between dimers and monomers can be seen in Supplementary Information (Table S5), as well as a time-dependent analysis of a parallel experiment (Figure S3). (**B**) Schematic illustration of the reaction between a S-nitrosylated cysteine and a thiolate. (**C**) Mass spectrometric analysis of fully reduced TTR-V30M. (**D**) Mass spectrometric analysis of TTR-V30M incubated with 100 µM GSNO, illustrating the + 30 Da adduct from NO and around 50% labelling. (**E**) TTR-V30M incubated with 100µM H_2_O_2_, resulting in oxidation of the Cys10 thiol to sulfinic acid and single oxidation of Met13 and Met30, thus corresponding to a total mass increase of + 64 Da compared with the fully reduced form. Mass spectrometry was performed on the LC/MS TOF 6230B, (Agilent, Santa Clara, CA, USA) using the MassHunter Qualitative Analysis 12.0 software (Agilent, Santa Clara, CA)

The requirement of an oxidative agent is implicated in the formation of dsNNTTR, and we have previously shown that it can be obtained using both the oxidizing agent diamide as well as via disulfide shuffling of disulfide conjugated adducts [22]. However, not all forms of oxidation may catalyse the formation of a disulfide bond. Strong oxidants such as hydrogen peroxide (H_2_O_2_) instead readily modify protein thiols beyond reversible redox states, to form sulfinic (-SO_2_H) and sulfonic acids (-SO_3_H), thus preventing conversion into the disulfide-linked pathological species dsNNTTR. Mild oxidative agents such as the reactive nitrogen species (RNS) are therefore potentially more relevant from a mechanistic point of view in vivo, as they modify thiols in a more controlled and often reversible manner. Nitrosylating agents derived from nitric oxide can transiently form S-nitrosothiols (R-SNO), which act as activated intermediates that readily undergo further redox reactions, including disulfide bond formation. In vivo, such nitrosylation events occur continuously under conditions of inflammation and oxidative stress, even though the resulting adducts are typically short-lived and rapidly converted into downstream oxidation products. Based on this redox framework, we investigated whether nitrosative chemistry could facilitate disulfide-mediated TTR conversion by using S-nitrosoglutathione (GSNO) as a physiological S-nitrosylating agent. GSNO efficiently promotes transient thiol modification and serves as a donor of nitrosyl equivalents [64]. Excess of GSNO was then removed through a buffer exchange. The results show that an NO adduct on the sulfhydryl group effectively drives intermolecular disulfide bond formation in TTR, converting native protein into the dsNNTTR species, see Fig. 4, which indicates both the effect of S-nitrosylation as well as oxidation using H_2_O_2_. Together, these findings indicate that while strong oxidants, such as H_2_O_2_, favor irreversible thiol overoxidation and suppress disulfide chemistry, reactive nitrogen species provide a physiologically relevant pathway for disulphide cross-linking of TTR.

## Discussion

### High Pyroglutamic acid indicates a disturbed GSH homeostasis in symptomatic TTR-V30M gene carriers

In a recent study, we demonstrated that the formation of the aggregation-prone NNTTR species, initially discovered in plasma from TTR-V30M gene carriers [21], is promoted by the formation of a disulfide bridge [22]. This finding highlighted the redox environment as a potentially important factor in the development of TTR amyloidosis by influencing the conversion of native TTR and NNTTR [22]. The precise anatomical compartment in which dsNNTTR formation occurs remains to be established. However, the effect of oxidative and nitrosative stress is not restricted to the intracellular environment, as reactive species can cross cellular membranes and influence extracellular proteins. In the circulation, native TTR is likely protected by macromolecular crowding [22], whereas this stabilizing effect is reduced in the interstitial fluid. We therefore propose that tissue-associated oxidative stress may promote conversion of circulating TTR into dsNNTTR locally within the interstitial compartment, rather than exclusively during intracellular synthesis or processing. This interpretation is also consistent with the clinical efficacy of liver transplantation, where replacement of the main site of TTR production reduces disease progression despite continued peripheral tissue expression of TTR.

In vivo, oxidative conditions are frequently generated by ROS and RNS, which are normally tightly regulated by multiple enzymatic systems, among which GSH and GSH-dependent enzymes play central roles [39]. PGA is a degradation product of glutathione metabolism within the γ-glutamyl cycle and is directly associated with disruptions in GSH homeostasis and GSH depletion [44, 45, 65, 66]. In the present study, we show that plasma PGA levels in symptomatic TTR-V30M gene carriers are increased by approximately 32% compared with healthy controls (*p* < 0.0001). A similar difference was observed when symptomatic patients were compared with asymptomatic carriers, with an approximately 25% increase in PGA levels (*p* < 0.05). In contrast, asymptomatic TTR-V30M carriers did not differ significantly from healthy TTR wild-type controls, indicating that elevated PGA correlates with disease manifestation (Table 1).

Disturbances in glutathione metabolism can arise through multiple mechanisms. Clinical conditions associated with glutathione depletion include increased oxidative stress and dysfunction of enzymes within the γ-glutamyl cycle. Acquired PGA elevation can also occur during chronic acetaminophen use [67, 68] as acetaminophen metabolism consumes glutathione through conjugation reactions, thereby depleting GSH stores and increasing metabolic flux toward PGA formation [69]. Similarly, oxidative stress states such as sepsis and critical illness can predispose to pyroglutamate accumulation due to increased GSH demand driven by elevated reactive oxygen species production [65]. These conditions may also coincide with nutritional deficiencies, particularly of cysteine and glycine, which are required for glutathione synthesis, further impairing the cycle and promoting diversion toward PGA production [70, 71]. Malnutrition is therefore a recognized risk factor, as limited availability of glutamate, cysteine, and glycine restricts effective glutathione synthesis. Among these amino acids, cysteine is especially sensitive to oxidative stress [72], which may further limit its availability and contribute to PGA accumulation.

In addition to acquired causes, inherited defects in enzymes such as glutathione synthetase [73, 74] and 5-oxoprolinase result in markedly elevated pyroglutamate levels due to impaired glutathione synthesis. Although these disorders are very rare, they underscore the fundamental link between disrupted glutathione homeostasis and pyroglutamate accumulation [75].

Maintaining adequate GSH levels may therefore represent a potential therapeutic approach to delay disease onset or suppress progression.

### TTR-V30M amyloidosis is associated with inflammation, linking the disease indirectly to oxidative stress

Although oxidative stress in vivo may arise through several different routes, it is frequently linked to inflammation. TTR amyloidosis has previously been shown to correlate with inflammation, including increased levels of TNF-α, IL-1β, IL-8, IL-33, IFN-β, and IL-10 [76]. In addition, in vitro studies have shown that recombinant TTR amyloid aggregates are internalized by human cardiac fibroblasts, which subsequently secrete IL-6 and IL-8, providing mechanistic evidence that TTR aggregates can trigger inflammatory responses in cardiac cells [77].

The present study supports these findings, as we show that an increased kynurenine/tryptophan (Kyn/Trp) ratio, a well-established marker of inflammation [63, 78, 79], correlates with manifest disease. An increased Kyn/Trp ratio reflects increased *IDO1* activity, which can be induced by pro-inflammatory cytokines, including interferon-γ (IFN-γ), IL-6, and IL-1β [80].

Given that an increase, but not a decrease, in the Kyn/Trp ratio is expected to be associated with inflammation, and that we observe a one-directional trend towards increased values among the asymptomatic V30M carriers compared to controls (Table 2; Fig. 3B), we performed a one-tailed analysis. In this one-tailed directional analysis, a small increase in the Kyn/Trp ratio was observed between controls and asymptomatic TTR-V30M carriers; however, the corresponding two-tailed comparison did not reach statistical significance. Although still exploratory, these findings suggest that the Kyn/Trp ratio may warrant further evaluation as a potential marker for risk stratification in longitudinal studies. An inflammatory response among asymptomatic V30M carriers is also in accordance with a previous study [76].

Together, these results indicate that TTR-V30M amyloidosis is associated with systemic inflammatory activation, which may contribute to increased oxidative stress. The link between inflammation and manifested disease is at present only correlative, and the cause of the inflammatory response can at this point not be determined with certainty; however, aggregates of TTR, including oligomeric forms, have previously been linked to cytotoxicity [81–85] supporting the possibility that misfolded TTR may contribute to this response.

### Nitrosylation of cystein 10 promotes dsNNTTR formation

Our results demonstrate that nitrosative thiol modification efficiently promotes intermolecular disulfide bond formation in TTR, as evidenced by the prominent dsNNTTR dimer observed by non-reducing SDS–PAGE following GSNO treatment. GSNO was used solely as a nitrosylating agent to generate S-nitrosylated TTR, as previously shown [64], and excess reagent was removed prior to analysis.

In contrast, strong oxidative conditions induced by H₂O₂ resulted in irreversible oxidation. Our results further show that efficient conversion depends on a defined balance between free thiolate and nitrosylated cysteine species, consistent with a mechanism in which transient S-nitrosylation facilitates thiol–disulfide exchange. Titration experiments revealed that the optimal GSNO concentration corresponded to approximately 50% cysteine labeling, which is consistent with the reaction requiring both S-nitrosylated cysteine and free thiolate. A parallel time-dependent analysis showing the dsNNTTR-promoting effect of NO is presented in Supplementary Information, Figure S3. Although the effect of NO labelling is clear, the degree of labelling can vary somewhat between biological replicates, likely due to subtle differences in labelling conditions. This results in small differences in the overall yield of dsNNTTR between experiments. Supplemental Information Fig. S3B shows an independent repetition of the experiment, illustrating that the yield varies somewhat.

Exposure to H₂O₂ resulted in a + 64 Da mass shift, consistent with overoxidation of Cys10 to sulfinic acid, together with oxidation of two methionine residues in the sequence. These modifications irreversibly block disulfide formation. Together, these findings indicate that reactive nitrogen species promote TTR crosslinking through reversible thiol activation, whereas strong oxidants divert cysteine chemistry toward stable overoxidized states that suppress disulfide-mediated conversion. The results highlight nitrosative redox chemistry as a potentially physiologically relevant pathway for dsNNTTR formation and support a model in which controlled thiol modification, rather than generalized oxidative stress, may contribute to pathological TTR crosslinking in vivo.

### A combined potential biomarker model with Pyroglutamic acid and Kyn/Trp ratio for TTR V30M risk prognosis

Peripheral plasma pyroglutamate (PGA) is a well-established biomarker of disrupted glutathione (GSH) homeostasis [65], whereas increased indoleamine 2,3-dioxygenase 1 *(IDO1*) activity, reflected by the kynurenine/tryptophan ratio, is a recognized marker of chronic inflammatory activation [61].

In the present study, elevated PGA levels were observed among 89% of symptomatic TTR-V30M patients and in 63% of asymptomatic carriers relative to the median of healthy controls (Table 1). ROC analysis yielded an AUC of 0.79 for patient/control comparisons and 0.63 for carrier/control comparisons, indicating progressive disruption of glutathione homeostasis across disease stages. Consistently, a higher proportion of patients compared with gene carriers exhibited elevated PGA levels (74%).

Kyn/Trp values above the control median were observed among 83% of patients and 80% of asymptomatic carriers (Table 2) compared to the median of healthy controls. The high prevalence among carriers likely reflects their age (≥ 55 years), which corresponds to the typical onset window for TTR-V30M amyloidosis in Sweden. This may suggest that a subset of carriers may already exhibit early disease-associated changes despite not fulfilling current diagnostic criteria. ROC analysis yielded AUC values of 0.74 for patients versus controls and 0.62 for carriers versus controls (Supplemental Information, Table S4).

Combining PGA and Kyn/Trp ratio improved discriminatory performance, yielding an AUC of 0.84 for patient/control comparisons (Supplemental Information, Fig. S1A) and 0.70 for carrier/control comparisons (Supplemental Information, Fig. S1B). These values indicate moderate discriminatory power and support the potential combined use of these markers for risk assessment.

The relatively low AUC of 0.64 for patient/carrier comparisons likely reflects overlap in age distribution, with many carriers already within the disease onset window, Supplemental Information Fig. S1C. Future studies focusing on younger asymptomatic carriers and age-matched controls may improve stratification and enable earlier detection of disease progression.

## Conclusion

We show that plasma PGA levels are elevated in symptomatic TTR-V30M carriers compared with asymptomatic carriers and healthy controls. As PGA is linked to perturbations in the γ-glutamyl cycle and glutathione metabolism, these findings are consistent with reduced antioxidant reserve or increased antioxidant demand in affected individuals.

In parallel, the increased Kyn/Trp ratio in symptomatic patients supports the presence of inflammatory activation. Together, these metabolic alterations suggest a biochemical framework in which inflammation, oxidative stress, and TTR misfolding may be interconnected in TTR-V30M amyloidosis.

**Fig. 5:**
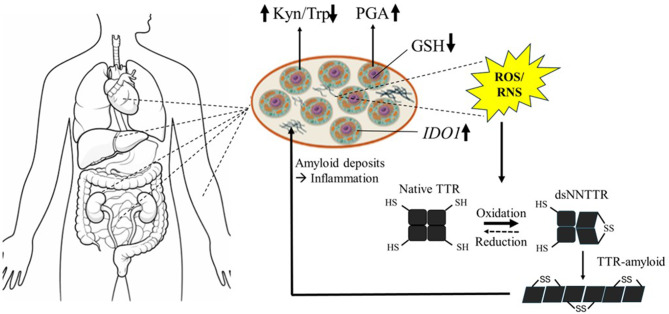
Schematic illustration of the link between oxidative stress, inflammation, and the conversion of native TTR into dsNNTTR and amyloid

Altered glutathione homeostasis and associated redox imbalance may therefore represent potential therapeutic areas for further investigation. Strategies aimed at restoring glutathione homeostasis or reducing oxidative and inflammatory stress could also be explored as possible complements to existing TTR-targeted therapies. In addition, PGA and Kyn/Trp may have potential as biomarkers for disease monitoring and risk stratification, although their clinical utility will require validation in independent and longitudinal cohorts. A schematic illustration of our findings and a summary of the proposed hypothesis are shown in Fig. 5.

### Limitations

It should be noted that the present cohort represents a late-onset Swedish TTR-V30M population, which may differ from populations in other endemic regions. Environmental factors, including diet and micronutrient availability, may influence disease penetrance and should therefore be considered when interpreting these findings. Further studies are thus warranted to determine the role of disrupted glutathione homeostasis in disease initiation and progression.

### Ethical permits

The procurement of human plasma samples was approved by the Swedish Ethical Review Authority (approval numbers 2022–04317–04 and 2022–06333–02). Blood samples from TTR-V30M mutation carriers and age-matched TTR wild-type controls were collected in EDTA-containing tubes. Plasma was separated and stored at − 80 °C within one hour of collection. Each sample was thawed only once prior to analysis. All procedures involving human participants were conducted in accordance with the principles of the Declaration of Helsinki.

## Supplementary Information

Below is the link to the electronic supplementary material.

Supplementary Material 1

## Data Availability

The analyzed data supporting the findings of this study are available within the article and its Supplementary Information file. The raw targeted metabolomics datasets are available from the corresponding author upon reasonable request.

## Declarations

### Authors note

These results were presented in parts at the Metabolomics in Life Science conference, 27–28 January 2026, Umeå, Sweden.

### Use of artificial intelligence

The authors acknowledge the use of Microsoft Copilot AI to generate the eukaryotic cell image and the image of a human body used as a component in Fig. 5.

### Links

https://www.kegg.jp/pathway/hsa00480, https://www.kegg.jp/entry/hsa00380, https://www.metaboanalyst.ca/home.xhtml

## Abbreviations

ANOVA: Kruskal–Wallis analysis of variance
ATTR: Transthyretin amyloidosis
AUC: Area under the curve
CV: Coefficient of variance
dsNNTTR: Aggregation-prone TTR species
DPD: 99mTc-DPD, 3,3-diphosphono-1,2-propanodicarboxylic acid
GSH: Glutathione
GSSG: Glutathione disulphide
GCL: γ-glutamylcysteine synthetase / glutamate–cysteine ligase
GPx: Glutathione peroxidase
GSNO: S-nitrosylglutathion
*IDO*: Indoleamine 2,3-dioxygenase 1
IQR: Interquartile range
IS: Internal standard
KEGG: Kyoto Encyclopedia of Genes and Genomes
Kyn/Trp: Kynurenine/tryptophan
LC/MS TOF: Liquid chromatography–time-of-flight mass spectrometry
LOD: Limit of Detection
LOQ: Limit of Quantitation
MRM: Multiple Reaction Monitoring
NNTTR: Non-native fold of TTR
NPH: Nitrophenyl hydrazine
PGA: Pyroglutamic acid
*p*: Mann–Whitney U test significance score
RNS: Reactive nitrogen species
ROC: Receiver Operating Characteristic
ROS: Reactive oxygen species
S/N: Signal-to-noise ratio
TCEP: Tris (2-carboxyethyl)phosphine
TTR: Transthyretin
