# Supplementary material for: Disrupted glutathione homeostasis in the pathogenesis of TTR-V30M amyloidosis
Source: Biomark Res. 2026 Jul 11;14:83. doi: 10.1186/s40364-026-00970-8 (PMC13366904; doi:10.1186/s40364-026-00970-8)
Supplement: Supplementary file 1 — Supplementary Material 1 [file 40364_2026_970_MOESM1_ESM.pdf]

Supplemental material

Table S1: Age and gender and status details for individuals included in the analysis
Status: C: Healthy controls (Wt TTR); **ASV30M:** AsymptomaticV30M gene carriers; **V30M:** Patients/ symptomatic V30M gene carriers.

Control(n)=26	Male:16	Female:10		ASV30M(n)=30; Male:10; Female: 20				V30M(n)=47; Male:30; Female: 17			
**Subject ID**	**Gender**	**Status**	**Age**	**Subject ID**	**Gender**	**Status**	**Age**	**Subject ID**	**Gender**	**Status**	**Age**
**P807**	M	C	74	**P853**	F	ASV30M	85	**P758**	M	V30 M	83
**P808**	F	C	67	**P854**	M	ASV30M	82	**P763**	M	V30 M	80
**P809**	M	C	66	**P855**	F	ASV30M	79	**P764**	M	V30 M	78
**P810**	F	C	59	**P857**	F	ASV30M	77	**P766**	M	V30 M	79
**P811**	M	C	57	**P858**	F	ASV30M	76	**P772**	M	V30 M	74
**P812**	M	C	57	**P859**	F	ASV30M	73	**P785**	M	V30 M	71
**P813**	M	C	56	**P860**	M	ASV30M	71	**P787**	F	V30 M	69
**P814**	M	C	56	**P861**	M	ASV30M	70	**P793**	M	V30 M	65
**P815**	F	C	55	**P862**	F	ASV30M	68	**P794**	F	V30 M	64
**P816**	M	C	55	**P863**	M	ASV30M	68	**P796**	M	V30 M	61
**P830**	M	C	88	**P864**	M	ASV30M	67	**P800**	M	V30 M	56
**P831**	F	C	80	**P865**	F	ASV30M	67	**P801**	M	V30 M	56
**P832**	F	C	77	**P866**	F	ASV30M	67	**P757**	F	V30 M	85
**P833**	M	C	74	**P868**	M	ASV30M	64	**P762**	F	V30 M	79
**P834**	M	C	67	**P869**	M	ASV30M	64	**P765**	M	V30 M	79
**P835**	M	C	65	**P870**	F	ASV30M	64	**P773**	M	V30 M	76
**P836**	M	C	63	**P871**	F	ASV30M	61	**P774**	F	V30 M	74
**P837**	M	C	58	**P873**	F	ASV30M	63	**P775**	F	V30 M	73
**P894**	F	C	71	**P874**	M	ASV30M	62	**P776**	M	V30 M	73
**P895**	F	C	65	**P875**	F	ASV30M	58	**P778**	M	V30 M	73
**P896**	F	C	63	**P876**	F	ASV30M	59	**P780**	M	V30 M	70
**P897**	M	C	61	**P877**	F	ASV30M	59	**P781**	M	V30 M	71
**P898**	M	C	59	**P878**	F	ASV30M	60	**P783**	M	V30 M	70

Table 1. continued.

**P899**	**F**	**C**	**58**	**P879**	**M**	**ASV30M**	**57**	**P784**	**M**	**V30 M**	**71**
**P900**	M	C	55	**P880**	F	ASV30M	58	**P789**	F	V30 M	69
**P901**	F	C	55	**P881**	F	ASV30M	58	**P790**	M	V30 M	66
				**P882**	F	ASV30M	56	**P791**	F	V30 M	69
				**P883**	F	ASV30M	57	**P795**	M	V30 M	61
				**P884**	F	ASV30M	55	**P799**	F	V30 M	55
				P885	M	ASV30M	56	**P867**	M	V30 M	64
								**P872**	M	V30 M	62
								**P856**	M	V30 M	79
								**P760**	M	V30 M	81
								**P761**	F	V30 M	79
								**P767**	M	V30 M	78
								**P770**	M	V30 M	78
								**P779**	M	V30 M	71
								**P756**	M	V30 M	88
								**P759**	F	V30 M	82
								**P777**	M	V30 M	74
								**P786**	M	V30 M	71
								**P768**	F	V30 M	77
								**P769**	F	V30 M	78
								**P788**	F	V30 M	68
								**P792**	F	V30 M	65
								**P797**	F	V30 M	56
								**P798**	F	V30 M	56

Cohort Selection criteria

Protocol for distinction between patients/symptomatic V30M carrier and asymptomatic V30M carrier

V30M carriers were classified as symptomatic patients once they fulfilled the Swedish national diagnostic criteria for TTR-V30M amyloidosis: a positive genetic test for the V30M mutation together with an amyloid-positive abdominal fat tissue biopsy and/or positive 99mTc-diphosphono-1,2-propanodicarboxylic acid (99mTc-DPD) scintigraphy in cases of suspected cardiac involvement.

Asymptomatic carriers were defined as individuals who tested positive for the V30M mutation but did not fulfil the additional diagnostic criteria for amyloid deposition. Genetic testing was performed by Sanger sequencing at Clinical Genetics, Umeå University Hospital, Sweden. The presence of the V30M mutation was assessed by sequencing the four exons of the TTR gene.

Amyloid deposition in biopsied tissue was confirmed by histological staining with Congo red and analysis using polarized light microscopy at the regional hospital, Norrland University Hospital, Umeå, Region Västerbotten, Sweden. Genetic testing and histological analyses were performed according to Swedish national guidelines.

Individuals who fulfilled the criteria described above were included as ATTR-V30M patients, whereas asymptomatic TTR-V30M carriers were included based on a positive TTR-V30M genotype and an amyloid-negative abdominal fat tissue biopsy.

Cohort characteristics

All participants were instructed to fast overnight for 12 hours, and plasma was collected in the morning. This procedure was used to minimize potential interference from diet and medication. None of the participants were classified as malnourished based on BMI and albumin status. Prior to inclusion, participants were asked whether they currently had, or had previously had, any liver- or kidney-related conditions. Only individuals who reported no history of either condition were selected as final plasma donors.

Table S2: Column statistics Results for PGA, Kynurenine and Tryptophan

The statistical analysis for metabolites pyroglutamic acid (PGA), kynurenine and tryptophan were performed using column statistics option provided by GraphPad Prism 5 software (San Diego, California, USA).

Metabolites	Group	Control (Wt TTR)	Gene Carrier (ASV30M)	Patients (V30M)
	**Samples per group**	26	30	47
**Pyroglutamic Acid**	**Minimum (µMol/l)**	9.32	11.62	11.07
	**25% Percentile(µMol/l)**	12.58	13.16	14.77
	**Median (µMol/l)**	14.12	14.89	18.62
	**75% Percentile (µMol/l)**	16.47	18.66	20.23
	**Maximum (µMol/l)**	18.33	47.64	37.96
	**Mean (µMol/l)**	14.38	17.06	18.24
	**Std. Deviation (µMol/l)**	2.38	6.94	4.64
	**Std. Error (µMol/l)**	0.46	1.26	0.67
	**Coefficient of variance (%)**	16.55%	40.67%	25.43%
	**Lower 95% CI (µMol/l)**	13.41	14.47	16.87
	**Upper 95% CI (µMol/l)**	15.34	19.65	19.60
**Kynurenine**	**Minimum (µMol/l)**	4.849	4.394	4.894
	**25% Percentile(µMol/l)**	5.876	6.206	6.959
	**Median (µMol/l)**	7.195	7.046	8.313
	**75% Percentile (µMol/l)**	9.092	8.174	9.866
	**Maximum (µMol/l)**	11.85	13.06	15.09
	**Mean (µMol/l)**	7.554	7.256	8.610
	**Std. Deviation (µMol/l)**	1.847	1.641	2.129
	**Std. Error (µMol/l)**	0.3623	0.2997	0.3105
	**Coefficient of variance (%)**	24.45%	22.61%	24.72%
	**Lower 95% CI (µMol/l)**	6.808	6.643	7.986
	**Upper 95% CI (µMol/l)**	8.300	7.869	9.235

Table S2. continued.

Tryptophan	Minimum (µMol/l)	36.15	30.53	33.92
	25% Percentile (µMol/l)	44.30	41.76	44.09
	Median (µMol/l)	54.78	45.79	48.54
	75% Percentile (µMol/l)	63.30	53.10	54.38
	Maximum (µMol/l)	76.72	63.72	71.53
	Mean (µMol/l)	54.77	46.99	49.02
	Std. Deviation (µMol/l)	11.65	7.251	7.750
	Std. Error (µMol/l)	2.285	1.324	1.130
	Coefficient of variance (%)	21.27%	15.34%	15.80%
	Lower 95% CI (µMol/l)	50.06	44.28	46.74
	Upper 95% CI (µMol/l)	59.48	49.70	51.29
IDO1 (Kynurenine/ Tryptophan)	Minimum	0.09718	0.09672	0.1038
	25% Percentile	0.1095	0.1337	0.1389
	Median	0.1337	0.1495	0.1674
	75% Percentile	0.1593	0.1743	0.2145
	Maximum	0.2027	0.2953	0.3215
	Mean	0.1407	0.1567	0.1793
	Std. Deviation	0.03246	0.03820	0.04998
	Std. Error	0.006367	0.006975	0.007213
	Coefficient of variance (%)	23.07%	24.37%	27.87%
	Lower 95% CI	0.1276	0.1424	0.1648
	Upper 95% CI	0.1538	0.1710	0.1938

Table S3. Statistical analysis Anova with post hoc test and Mann-Whitney U test table for analyzed metabolites. The analysis was performed using GraphPad Prism 5 software (San Diego, California, USA).

Metabolite	Control(Wt-TTR) – Carrier(ASV30M) - Patient(V30M)		Exact or approximate *p* value: Gaussian Approximation (95% confidence) — Mann-Whitney test								
			Control(Wt) - Carrier ASV30M)			Control(Wt) -Patient(V30M)			Carrier (ASV30M) -Patient(V30M)		
	Anova (*p*)	Kruskal-Wallis statistic	*p* value and significance (1 tailed)	*p* value and significance (2 tailed)	Mann-Whitney U	*p* value and significance (1 tailed)	*p* value and significance (2 tailed)	Mann-Whitney U	*p* value and significance (1 tailed)	*p* value and significance (2 tailed)	Mann-Whitney U
Pyroglutamic acid (PGA)	<0.0001	17.772	0.0564	0.1129	293	< 0.0001	< 0.0001	256	0.0074	0.0147	471
Kynurenine	0.0085	9.914	0.3376	0.6753	364	0.0188	0.0367	430	0.0014	0.0028	418
Tryptophan	0.0279	7.117	0.0067	0.0134	239	0.0204	0.0409	433	0.1567	0.3135	608
Kynurenine/ Tryptophan	0.0019	12.141	0.0477	0.0954	288	0.0004	0.0009	321	0.0297	0.0594	524

Table S4. Receiver operating characteristics table for multivariate (Random-forest) and univariate and biomarker validation for pyroglutamic acid and *IDO1*. For the Multivariate Biomarker validation part, the metabolite data were normalized with Log 2 followed by scaling using autoscaling feature (mean-centered and divided by the standard deviation of each variable). The normalization report can be obtained in Figure S2. https://www.metaboanalyst.ca/MetaboAnalyst/upload/RocUploadView.xhtml)

**Group**	**Receiver Operating Characteristic (ROC) Univariate**			**ROC Multivariate (Random forest)**	
	**Metabolite**	**AUC**	**95% Confidence limit**	**AUC**	**95% Confidence limit**
**Patient-V30M-Healthy Control**	**Pyroglutamic acid**	0.79	0.703-0.807	0.845	0.708-0.931
	***IDO1* (Kynurenine/ Tryptophan)**	0.745	0.626-0.852		
**Asymptomatic V30M Carrier-Healthy Control**	**Pyroglutamic acid**	0.639	0.486-0.762	0.709	0.523-0.865
	***IDO1* (Kynurenine/Tryptophan)**	0.624	0.493-0.802		
**V30M patient-AsymptomaticV30M carrier**	**Pyroglutamic acid**	0.665	0.539-0.784	0.636	0.504-0.790
	***IDO1* (Kynurenine/Tryptophan)**	0.628	0.509-0.741		

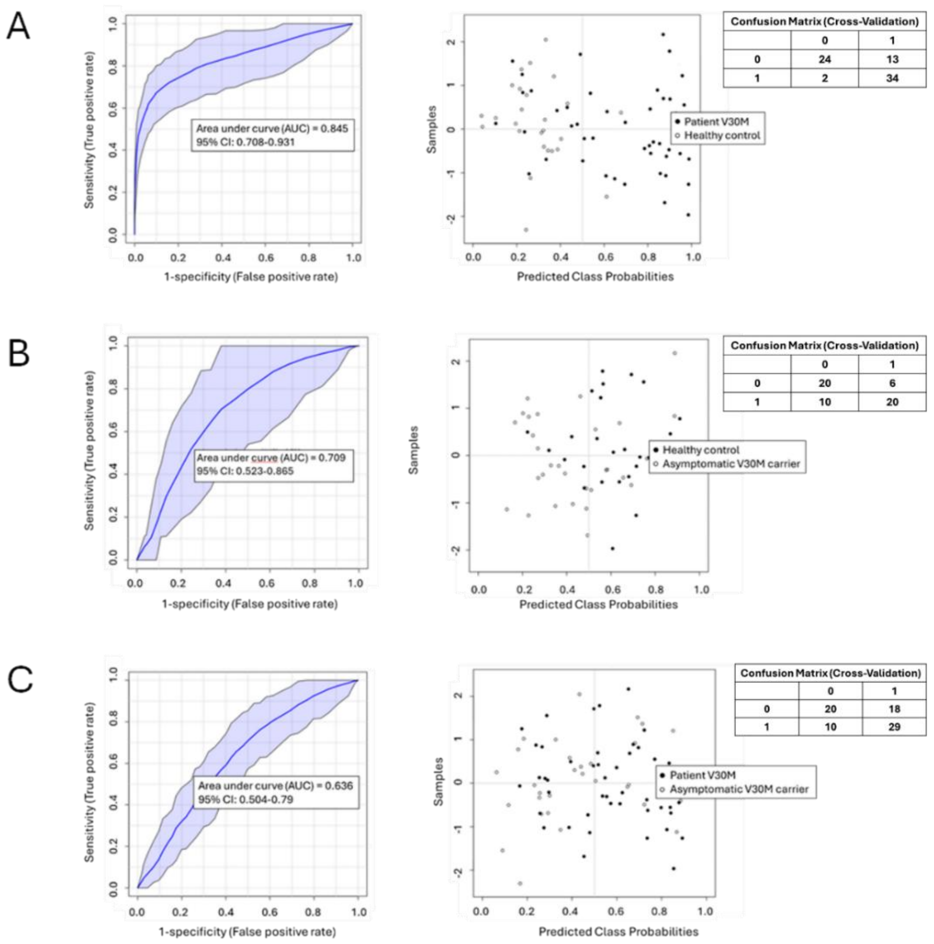

Figure S1. Receiver operating characteristics for multivariate biomarker validation between groups using Random-Forest technique with cross-validation probability and confusion matrix. (A) Patients V30M and Healthy control. **(B)** Asymptomatic V30M carrier and Healthy control. **(C)** Patient V30M and Asymptomatic V30M carriers. The figures had been created with MetaboAnalyst 6.0 biomarker validation tool, Tester mode.

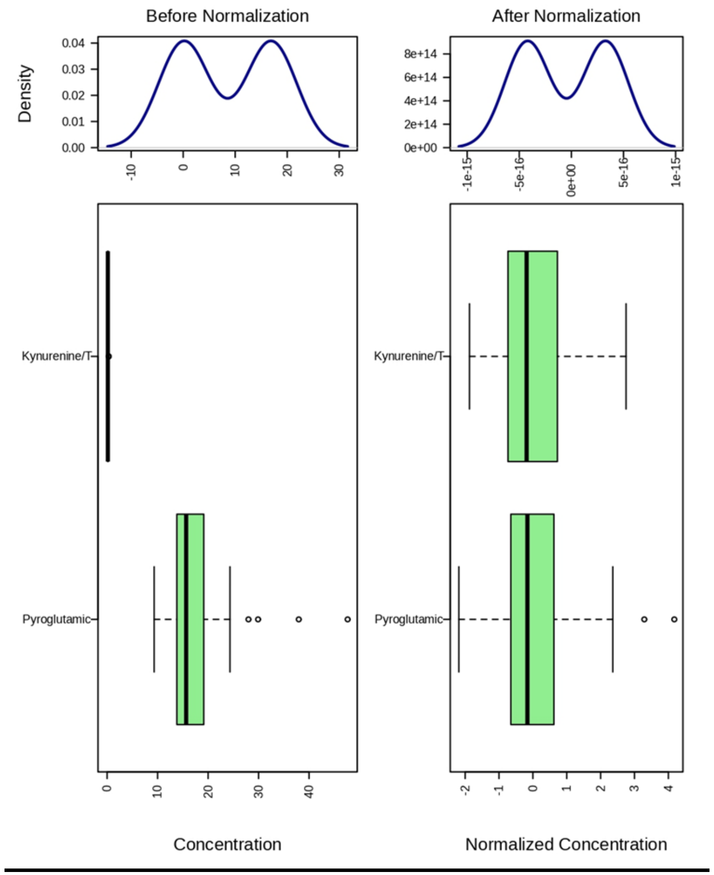

Figure S2. **Normalization for Biomarker validation and ROC with MetaboAnalyst 6.0.** Normalization is done by transforming the data with Log 2 and the autoscaling (mean-centered, divided by the standard deviation of each variable) function simultaneously during the data normalization step provided by the online tool.

Table S5. Densitometric analysis of the SDS-PAGE from Fig. 4A in the main article, illustrated as the dimer to monomer ratio. The analysis was performed using ImageJ 1.54g software (64-bit) for Windows (Wayne Rasband and Contributors, National Institutes of Health, USA). The SDS-PAGE analysis was repeated three times to verify the mechanism. The corresponding densitometric analysis displays a representative example.

**Sample**	**Dimer to monomer ratio**
**Lane 1: 0.1% H_2O_2**	0.018
**Lane 2: 100 µM BME**	0.017
**Lane 3: 0µM GSNO**	0.020
**Lane 4: 12.5 µM GSNO**	0.027
**Lane 5: 25 µM GSNO**	0.028
**Lane 6: 50 µM GSNO**	0.076
**Lane 7: 100 µM GSNO**	0.369
**Lane 8: 200 µM GSNO**	0.147
**Lane 9: 400 µM GSNO**	0.130

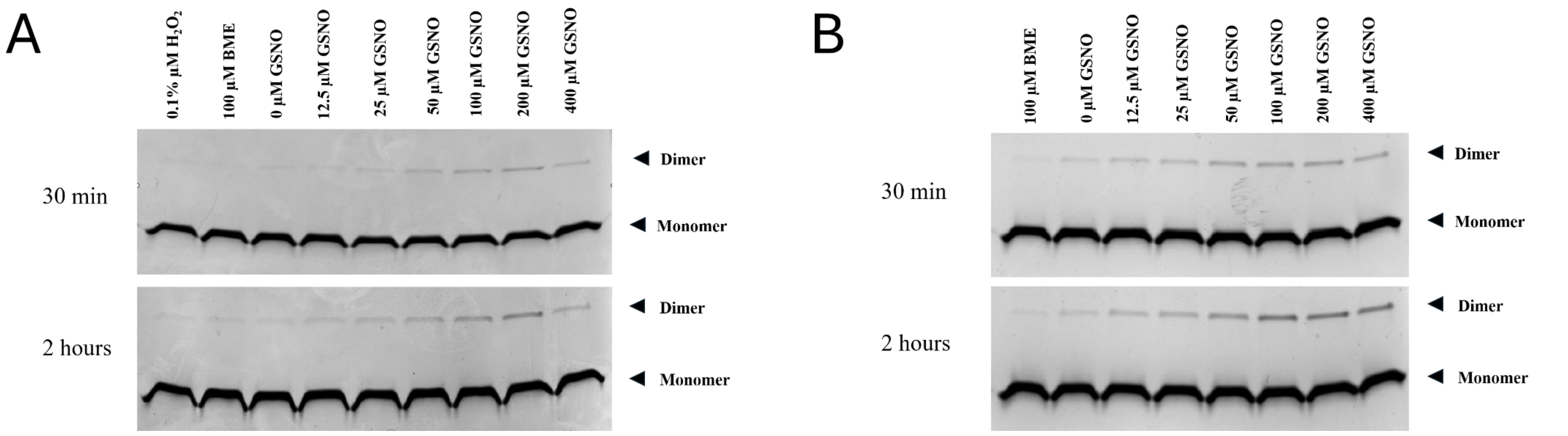

Figure S3. **Time-dependent formation of dsNNTTR as a function of NO conjugation. (A)** Recombinant TTR-V30M (8 µM) was treated with S-nitrosoglutathione (Sigma-Aldrich, St. Louis, MO, USA), in a serial dilution from 0 to 400 µM as described in the Materials and Methods. Samples were then incubated at room temperature for either 30 min or 2 hours, followed by analysis on SDS-PAGE. The result illustrates how conjugation with NO promotes the formation of dsNNTTR in a time and concentration-dependent manner. TTR-V30M treated with H2O2 and BME were included as controls. (B) Independent repetition of the effect of NO conjugation on TTR-V30M.
